# Validation of the Modified Multiplier of SES-CD (MM-SES-CD) to Predict Endoscopic Healing in Crohn’s Disease: A Post Hoc Analysis of the SEAVUE Trial

**DOI:** 10.1093/ibd/izaf137

**Published:** 2025-06-30

**Authors:** Dhruv Ahuja, Sama Anvari, Emily C L Wong, Parambir Dulai, John K Marshall, Vipul Jairath, Walter Reinisch, Neeraj Narula

**Affiliations:** Department of Medicine, Indira Gandhi Hospital, New Delhi, India; Department of Medicine (Division of Gastroenterology) and Farncombe Family Digestive Health Research Institute; McMaster University, Hamilton, ON, Canada; Department of Medicine (Division of Gastroenterology) and Farncombe Family Digestive Health Research Institute; McMaster University, Hamilton, ON, Canada; Division of Gastroenterology, Northwestern University, Chicago, IL, USA; Department of Medicine (Division of Gastroenterology) and Farncombe Family Digestive Health Research Institute; McMaster University, Hamilton, ON, Canada; Department of Medicine, Division of Gastroenterology, Western University, London, ON, Canada; Department of Internal Medicine III, Division of Gastroenterology and Hepatology, Medical University of Vienna, Währinger Gürtel 18-20, Vienna, Austria; Department of Medicine (Division of Gastroenterology) and Farncombe Family Digestive Health Research Institute; McMaster University, Hamilton, ON, Canada

**Keywords:** endoscopy, clinical trials, Crohn’s disease

## Abstract

**Background:**

The modified multiplier of the SES-CD (MM-SES-CD) has been shown to predict future endoscopic healing (EH) in patients with Crohn’s disease. The purpose of this study was to validate baseline MM-SES-CD categories of severity and determine their prognostic value for predicting 1-year EH.

**Methods:**

Participants in the SEAVUE trial (*n* = 386) were classified based on baseline endoscopic disease severity using MM-SES-CD cut-offs as mild (≥ 22.5 to < 31), moderate (≥ 31 to < 45), and severe (≥ 45) disease. The primary outcome was achieving 1-year endoscopic healing (EH) as measured by the MM-SES-CD score (< 22.5). Secondary outcomes included clinical and biochemical remission at 1 year based on patient-reported outcomes and fecal calprotectin (FCP)(< 250 mcg/g).

**Results:**

MM-SES-CD < 22.5 at 1 year was achieved in 62.0% of patients with baseline mild endoscopic disease, 48.6% with moderate disease, and 33.8% with severe disease (*P* < .001). A similar trend was observed for patient-reported outcome (PRO-2) clinical remission, which was reached in 78.9% of patients with baseline mild endoscopic disease, 72.9% of those with moderate, and 66.2% of those with severe disease (*P* = 0.09). The likelihood of fecal calprotectin (FCP) remission was significantly associated with baseline endoscopic disease severity (*P* = .008).

**Conclusion:**

Baseline MM-SES-CD-based cutoffs for endoscopic disease severity show prognostic value for the likelihood of achieving 1-year EH, PRO2 remission, and FCP remission. These findings suggest that the MM-SES-CD can be used both to measure baseline endoscopic disease severity and predict outcomes at 1 year in patients with moderate to severe CD.

**Key Messages**

- What is already known? The modified multiplier of SES-CD (MM-SES-CD) has been shown to better account for the prognostic implications of disease location and features in patients with Crohn’s disease compared to the SES-CD.
- What is new here? This post-hoc analysis of SEAVUE validates that baseline MM-SES-CD scores demonstrate prognostic value for achieving endoscopic healing, PRO2 remission, and fecal calprotectin remission at 1 year.
- How can this study help patient care? Using the MM-SES-CD to measure endoscopic disease activity can help improve prognostication of outcomes in patients with Crohn’s disease both within clinical trials and practice.

## Introduction

Crohn’s disease (CD) is a chronic inflammatory disease of the gastrointestinal tract that causes significant morbidity, disability, and diminished quality of life.^1^ In recent years, evidence has accumulated demonstrating the association between mucosal inflammation and long-term disease-related complications, even in the absence of clinical symptoms.^2,3^ As such, international treatment paradigms for patients with CD have shifted to emphasize the importance of achieving endoscopic healing (EH) to improve short and long-term outcomes such as maintaining clinical remission (CR), preventing relapse, and decreasing the need for surgeries.^4^

Several scoring systems exist to assess endoscopic disease burden in patients with CD. The most frequently used tool in clinical trials is the Simple Endoscopic Score for Crohn’s disease (SES-CD), which was created to simplify the more complicated Crohn’s Disease Endoscopic Index of Severity (CDEIS).^5,6^ The SES-CD assigns an equally weighted, linear scoring system for disease activity across all disease locations within the bowel that may not adequately capture the variable prognostic value of individual findings on EH and long-term outcomes.^7–9^ Indeed, one post-hoc analysis of the SONIC trial revealed that those with large ulcers in the rectum and ileum were less likely to achieve endoscopic remission, but the SES-CD does not take into account how disease features or location may impact the likelihood of healing.^8^ The modified multiplier of SES-CD (MM-SES-CD) was created to weigh the individual impact of disease characteristics, such as location, size, and extent of ulceration, therefore accounting for the variable prognostic value of these parameters for predicting response to therapy and EH.^10,11^ For example, scored elements such as ileal disease and stricturing are associated with a higher multiplier compared to the same findings in the transverse colon.^10^ It has previously been shown to more accurately predict endoscopic remission compared to the SES-CD in both training and testing datasets.^10^

Current guidelines generally define endoscopic healing as SES-CD < 3, but acknowledge that further research is required to better define targets for healing associated with positive long-term outcomes.^4,12^ Narula et al. recently proposed that an MM-SES-CD score of < 22.5 may be considered a treatment target for endoscopic healing. They demonstrated that achieving MM-SES-CD < 22.5 at 1 year while on active therapy best discriminated patients with CD who did not experience long-term disease progression, findings that were further validated in a prospective dataset with long-term patient follow-up.^13^

The MM-SES-CD has been shown to more accurately quantify endoscopic disease burden and predict the likelihood of EH at 1 year (defined as SES-CD < 3) than the SES-CD.^10^ However, further validation of suggested cutoffs for the classification of endoscopic disease severity and EH is required before incorporation within clinical trials or practice. Therefore, the purpose of this study was to use an independent dataset to validate prior findings suggesting that baseline MM-SES-CD scores can predict 1-year EH, using the alternative definition for endoscopic healing (MM-SES-CD < 22.5) proposed by Narula et al.

## Materials and Methods

This study is a post-hoc analysis of the SEAVUE trial using individual participant data in CD patients. SEAVUE was a randomized controlled trial which compared adalimumab and ustekinumab in biologic-naïve CD patients.^14^ Data that were previously collected were obtained through the Yale University Open Data Access (YODA) project (Yale Open Data Access #2023-5158) with permission from Janssen Inc.^15^ As this study did not involve human participation and utilized de-identified data, it was determined that additional informed consent and local ethics approval were not required.

### Study Protocol and Participants

SEAVUE’s study design and eligibility criteria have been described previously.^14^ Briefly, the trial enrolled biologic naïve patients with moderate-to-severe CD based on the Crohn’s Disease Activity Index (CDAI) scores ranging from 220 to 450 who had not responded to or were intolerant to corticosteroids and/or immunomodulators, or were corticosteroid dependent. It was a treat straight through design and patients were randomized to receive either ustekinumab or adalimumab. Endoscopy was performed at baseline and at 1 year and was read centrally using the SES-CD score. Trial participants had to have at least one ulcer of any size at baseline endoscopic evaluation which translates into an SES-CD score ≥ 3. In total, 386 subjects were included, of whom 191 and 195 patients received ustekinumab and adalimumab, respectively.

### Endoscopic Disease Severity Characterization

Baseline MM-SES-CD scores were derived post-hoc from the centrally read endoscopic parameters of the SES-CD using individual patient-level data. For this analysis, baseline endoscopic disease severity was categorized as mild (≥ 22.5 to < 31), moderate (≥ 31 to < 45), and severe (≥ 45), similar to what had previously been proposed in a prior study.^11^ Of note, the SEAVUE dataset was not utilized within the derivation of the MM-SES-CD categories study.^10^ A detailed calculation of MM-SES-CD parameters can be found online [https://www.mcmasteribd.com/mm-ses-cd] and has been previously published.^10^ It is also detailed in Table S1.

### Treatment Outcomes

Outcomes were assessed at the end of the maintenance period (1 year). The primary outcome was 1-year endoscopic healing defined as MM-SES-CD < 22.5. Secondary outcomes including 1-year patient-reported outcome (PRO-2) remission (defined as 7-day average of stool frequency sub-score ≤ 1.5 and abdominal pain mean daily score ≤ 1),^16^ endoscopic remission defined as SES-CD < 3, and fecal calprotectin remission (< 250 mcg/g) in those with elevated fecal calprotectin at baseline. Elevated fecal calprotectin was defined as ≥ 250 mcg/g.

### Statistical Analysis

With respect to this post-hoc analysis, among the 386 patients in SEAVUE, those with MM-SES-CD < 22.5 at baseline were excluded, as that was our primary outcome of interest.^13^ Patients with missing endoscopic data were also excluded. Continuous variables were described using means with standard deviations, and categorical variables were described as proportions. Chi-square tests of trend were used to compare categorical variables. Baseline characteristics were summarized by the baseline MM-SES-CD categories as means (standard deviation) or medians (range) for continuous variables and as frequency (%) for categorical variables.

Logistic regression was performed to assess the association of baseline categories of MM-SES-CD and 1-year endoscopic healing (MM-SES-CD < 22.5). Univariate analyses were conducted to evaluate the relationship between other variables of interest at baseline and achievement of 1-year MM-SES-CD < 22.5. Subsequently, multivariate logistic regression using backward stepwise selection was performed. Only those baseline variables with *P* < .10 on univariate analysis were considered for inclusion into the multivariate model. Variables had to have *P* < .05 to be selected for retention within the multivariate model. Unadjusted odds ratios (ORs) and adjusted ORs (aORs) were presented with associated 95% CIs.

To test the model performance for MM-SES-CD < 22.5 (endoscopic healing), areas under the receiver operator curve (AUC) were derived to test the discrimination aspect of the model. F1 score was calculated to study the precision and recall of the model when class distribution is asymmetric (scores range from 0 to 1, with a score of 0 suggesting either precision or recall is zero and 1 suggesting perfect precision and recall). Sensitivity, specificity, positive and negative predictive values were also calculated. All analyses were done using R version 4.3.0 (Vienna, Austria). Data are available upon request.

## Results

### Patient Characteristics

Patient baseline characteristics are summarized in Table 1. A total of 386 patients were enrolled in the trial. Of these, we excluded 163 (42.2%) patients who had inactive disease by the MM-SES-CD (MM-SES-CD < 22.5) and 11 patients with missing endoscopy data at baseline. Of the 212 patients included in the analyses, 71 (33.5%) had mild disease (MM-SES-CD ≥ 22.5 to < 31), 70 (33.0) had moderate disease (MM-SES-CD ≥ 31 to < 45), and 71 (33.5%) had severe disease (MM-SES-CD ≥ 45). The mean age of the cohort was 36.06 ± 13.05 years, and the cohort was 47.6% male. The analyzed subjects had a mean disease duration of 5.89 ± 8.6 years. At baseline, 137 (64.6%) patients had an elevated CRP (> 5.0) and 141 (66.5%) had an elevated fecal calprotectin (> 250 mcg/g). Patients classified as having severe disease were significantly more likely to have ileocolonic disease and had higher baseline levels of CRP and fecal calprotectin (FCP).

**Table 1.** Baseline characteristics of patients from the SEAVUE trial (*n* = 212) stratified by baseline MM-SES-CD based categories.

Variable	Mild (MM-SES-CD ≥ 22.5 to < 31) (*N* = 71)	Moderate (MM-SES-CD 31 to < 45) (*N* = 70)	Severe (MM-SES-CD ≥ 45) (*n* = 71)	*P* value
Age (mean (SD))	36.14 (13.29)	38.93 (13.15)	33.14 (12.21)	.02
Sex (males)	34 (47.9%)	31 (44.3%)	40 (56.3%)	.42
Race: White	65 (91.5%)	66 (94.3%)	62 (87.3%)	.52
Black	3 (4.2%)	2 (2.9%)	1 (1.4%)	
Asian	2 (1.8%)	1 (1.4%)	6 (8.5%)	
Others	1 (1.4%)	1 (1.4%)	2 (2.8%)	
Duration in years (mean (SD))	5.55 (7.35)	6.92 (9.95)	5.19 (8.26)	.47
Prior Surgery	24 (33.8%)	17 (24.3%)	12 (16.9%)	.14
Location: Colonic	10 (14.3%)	17 (25%)	14 (19.7%)	<.01
Ileal	25 (35.7%)	16 (23.5%)	10 (14.1%)	
Ileocolonic	35 (50%)	35 (51.5%)	47 (66.2%)	
Perianal disease	16 (22.5%)	21 (30.0%)	19 (26.8%)	.33
Active intervention:				<.01
Adalimumab	32	34	36	
Ustekinumab	39	36	35	
Upper GI disease	5 (7%)	7 (10%)	14 (19.7%)	.12
Baseline steroid usage	7 (9.8%)	8 (11.4%)	9 (12.7%)	.89
Baseline IMM usage	16 (22.5%)	6 (8.6%)	7 (9.9%)	.62
Current smoking	16 (22.5%)	18 (25.7%)	21 (29.6%)	.82
Baseline Hb (mean)	13.25 (1.72)	12.77 (1.55)	12.64 (1.84)	<.01
Baseline albumin (mean)	4.31 (0.41)	4.18 (0.45)	4.1 (0.45)	<.01
Baseline CRP (mean)	9.07 (11.59)	19.04 (31.48)	22.99 (22.80)	<.01
Baseline FCP (mean)	1005.01 (1067.33)	1657.33 (2080.02)	2505.76 (3149.64)	<.01
Total SES-CD score				<.01
Inactive (< 3)	0 (0%)	0 (0%)	0 (0%)	
Mild (3 – 6)	14 (19.7%)	0 (0%)	0 (0%)	
moderate (7-15)	57 (80.3%)	56 (80%)	11 (15.5%)	
Severe (> 15)	0 (0%)	14 (20%)	60 (84.5%)	

### Achievement of Endoscopic Healing, PRO-2 Remission, and Fecal Calprotectin Remission at 1 Year

**Figure 1.**
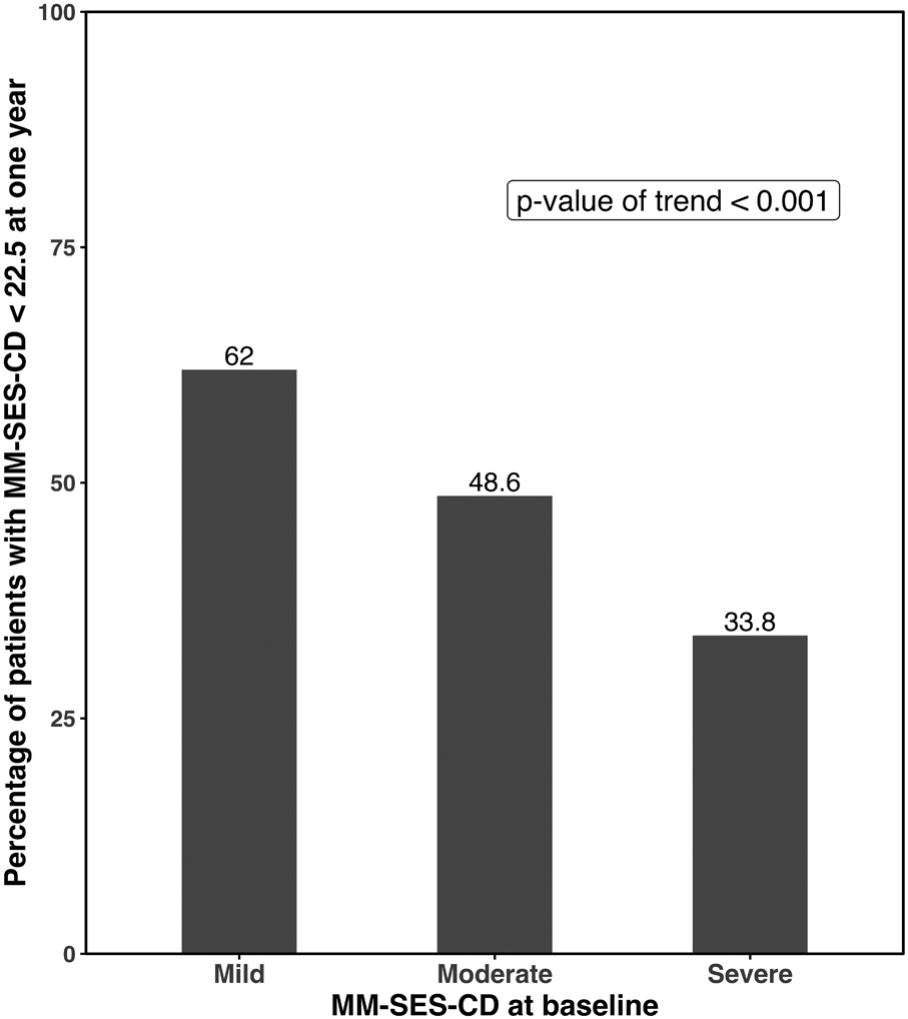
Percentage of patients with MM-SES-CD-based endoscopic healing (score < 22.5) at 1 year, stratified by baseline MM-SES-CD score categories (mild ≥ 22.5 to < 31, moderate ≥ 31 to < 45, severe ≥ 45).

**Figure 2.**
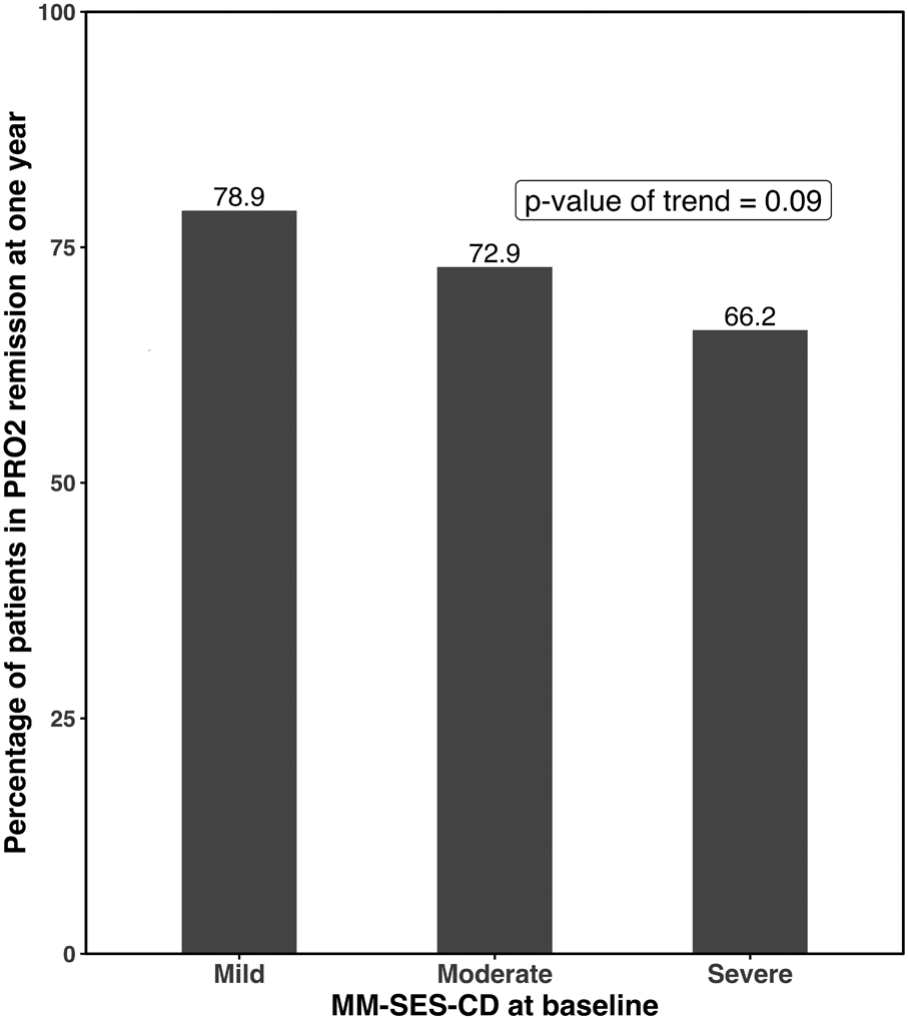
Percentage of patients in PRO2 remission at 1 year stratified by baseline MM-SES-CD categories (mild ≥ 22.5 to < 31, moderate ≥ 31 to < 45, severe ≥ 45).

**Figure 3.**
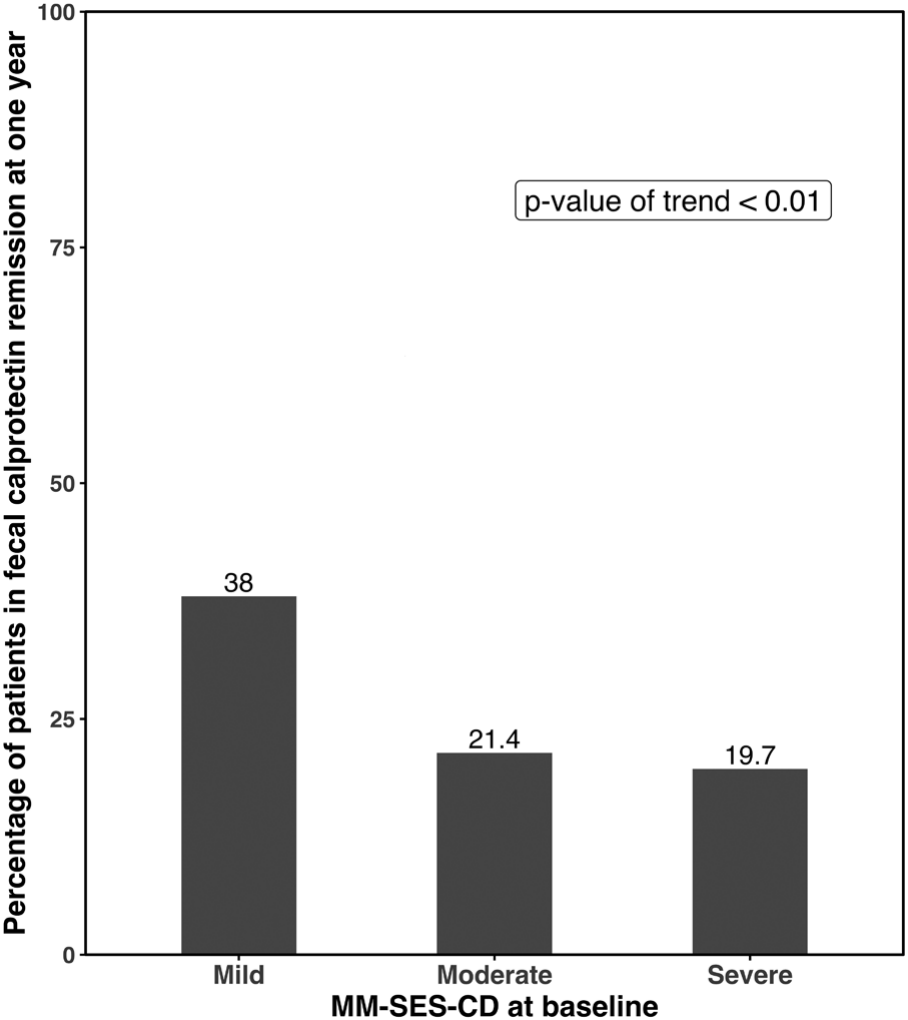
Percentage of patients with fecal calprotectin remission at 1 year stratified by baseline MM-SES-CD categories (mild ≥ 22.5 to < 31, moderate ≥ 31 to < 45, severe ≥ 45).

MM-SES-CD < 22.5 was achieved by significantly more patients with baseline mild endoscopic disease (44/71, 62%) as compared to those with moderate (34/70, 48.6%) and severe disease (24/71, 33.8%) [*P* < .001] as shown in Figure 1. Similar findings were observed for each biologic individually within subgroup analyses for adalimumab (Figure S1, *P* = .03) and ustekinumab (Figure S2, *P* = .01). When endoscopic remission was defined as SES-CD < 3, we also found that a larger proportion of patients with mild disease (21.1%) achieved remission compared to those with moderate (17.1%) and severe (12.7%) disease; however, this was not statistically significant (*P* = .13) (Figure S3). PRO2 remission was also assessed, as seen in Figure 2. There was a trend seen between baseline MM-SES-CD severity and PRO2 remission, as PRO2 remission was achieved by 56/71 (78.9%) patients with mild disease, 51/70 (72.9%) patients with moderate disease, and 47/71 (66.2%) patients with severe disease (*P* = .09). Similarly, FCP remission, defined as FCP ≤ 250 mcg/kg, was more likely to be seen in those with milder baseline endoscopic disease severity, and was observed in 18/48 (38%) patients with mild disease, as compared to 14/46 (21.4%) patients with moderate disease and 9/47 (19.7%) patients with severe disease (*P* = .008) (Figure 3).

### Univariate and Multivariate Model for Prediction of MM-SES-CD < 22.5 at 1 Year

In the univariate analysis of baseline variables, several were evaluated, and those found to have an association with the outcome of 1-year MM-SES-CD < 22.5 with *P* < .10 were considered for inclusion in the multivariate model (Table S2). In the univariate analysis, patients with severe MM-SES-CD had significantly lower odds of achieving 1-year MM-SES-CD < 22.5 as compared to those with mild disease (aOR 0.17, 95% CI 0.07-0.38, *P* < .001). Those with moderate disease also had significantly lower odds of achieving MM-SES-CD < 22.5 at 1 year (OR 0.39, 95% CI 0.16-0.92, *P* = .034) compared to those with mild disease (Table 2). A similar trend was noted when endoscopic remission was defined as SES-CD < 3, but no significant differences in odds ratios were observed between the mild, moderate, and severe groups (Table S3). We did not find a significant association between the treatment arm (adalimumab vs ustekinumab) and the likelihood of achieving MM-SES-CD < 22.5 at 1 year (p = 0.14) (Supplementary Table 2). In addition to baseline MM-SES-CD severity, baseline albumin, FCP, and presence of upper GI disease or ileal disease had an association with 1 year MM-SES-CD < 22.5 and were considered for inclusion within the multivariate model. None of the covariates were considered significant (*P* > .05) in the backward selection of covariates. For the outcome of PRO2 remission, univariate screening of covariates revealed a significant association of age, baseline albumin, and the type of active intervention. Only age was considered as a covariate through the backward selection process and added to the multivariable model. Patients with severe endoscopic disease had significantly lower odds of achieving PRO2 remission at 1 year compared to those with mild disease in the multivariate analysis (aOR 0.43, 95% CI 0.19-0.94, *P* = .04). However, no significant difference was observed in patients with moderate disease (aOR 0.79, 95% CI 0.35-1.77, *P* = .5; Table 3). Finally, on univariate analysis, patients with severe endoscopic disease had significantly lower odds of achieving FCP remission compared to those with mild disease (OR = 0.36, 95% CI 0.15-0.85, *P* = .02), whereas the odds ratio for patients with moderate endoscopic disease did not achieve statistical significance (Table S4). In addition to baseline MM-SES-CD category, only baseline FCP had a significant association with FCP remission at 1 year on univariate analysis, but was not considered significant in backward selection and thus was not included for adjustment.

**Table 2.** Association between baseline MM-SES-CD and 1-year MM-SES-CD based endoscopic healing (< 22.5), *n* = 165.

	OR	95% CI	*P*- value
**Univariate analysis**			
Mild (MMSESCD ≥ 22.5 and < 31)	Reference	Reference	Reference
Moderate (MMSESCD ≥ 31 and < 45)	0.39	0.16-0.92	.034
Severe (MMSESCD ≥ 45)	0.17	0.07-0.38	<.001

**Table 3.** Association between baseline MM-SES-CD and 1-year PRO2 remission, *n* = 212.

	OR	95% CI	*P*- value
**Univariate analysis**			
Mild (MMSESCD ≥ 22.5 and < 31)	Reference	Reference	Reference
Moderate (MMSESCD ≥ 31 and < 45)	0.72	0.33-1.56	.41
Severe (MMSESCD ≥ 45)	0.52	0.24-1.1	.09
**Multivariate analysis**			
Mild (MMSESCD ≥ 22.5 and < 31)	Reference	Reference	Reference
Moderate (MMSESCD ≥ 31 and < 45)	0.79	0.35-1.77	.57
Severe (MMSESCD ≥ 45)	0.43	0.19-0.94	.04
Age	0.96	0.93-0.98	<.01

### Performance of Model of Achieving MM-SES-CD < 22.5 Based on Baseline Categories of MM-SES-CD

The predictive model evaluating the association between baseline MM-SES-CD categories and the likelihood of achieving 1-year endoscopic healing (MM-SES-CD < 22.5) from this validation cohort demonstrated moderate performance (Table 4). Specifically, the model’s ability to predict 1-year endoscopic healing was reflected in an AUC-ROC of 0.69 (95% CI: 0.58-0.79), indicating moderate discriminative capacity. The F1 score was 0.55, representing a balance between precision and recall in predicting remission. For the outcome of 1-year endoscopic healing, the model demonstrated a sensitivity of 52.4% (95% CI: 36.7-92.3), identifying just over half of the cases with endoscopic healing, and a specificity of 76.5% (95% CI: 31.6-85.7), correctly identifying non-achievement of endoscopic healing in over three-quarters of those cases. The positive predictive value for endoscopic healing was 57.9% (95% CI: 38.9-75), while the negative predictive value was 72.2% (95% CI: 60.5-83.3).

**Table 4.** Measures of model performance for association of baseline MM-SES-CD-based categories with 1-year MM-SES-CD remission.

Performance Measure	Validation Cohort
AUC-ROC	0.69 (0.58-0.79)
F1 score	0.55
Sensitivity (95% CI)	52.4 (36.7-92.3)%
Specificity (95% CI)	76.5 (31.6-85.7)%
Positive predictive value (95% CI)	57.9 (38.9-75)%
Negative predictive value (95% CI)	72.2 (60.5-83.3)%
Youden Index	0.29

## Discussion

This post-hoc analysis of SEAVUE data demonstrated that baseline categories of MM-SES-CD are predictive of 1-year endoscopic healing in patients with CD. We found that patients with moderate or severe baseline MM-SES-CD scores had lower odds of achieving EH as defined by an MM-SES-CD threshold of < 22.5 at 1 year compared to those with milder disease. Similarly, we observed lower 1-year rates of PRO2 remission in those with higher baseline MM-SES-CD scores, as patients with severe MM-SES-CD at baseline had less than half the odds of achieving PRO2 remission than those with mild scores (aOR 0.43, *P* = .04). Higher baseline MM-SES-CD scores were also associated with significantly reduced odds of achieving fecal calprotectin remission, with only 19.7% of individuals with severe disease achieving FCP remission at 1 year (*P* = .008). Overall, this study demonstrates that baseline categorization of endoscopic disease with the MM-SES-CD has prognostic utility given its association with 1-year EH, PRO2 clinical remission, and fecal calprotectin remission, which may have implications for both clinical trials and practice.

As collective understanding of the natural history of CD has evolved, therapeutic goals have expanded to include clinical, biochemical, and endoscopic targets. Current guidelines utilize the SES-CD or CDEIS to define endoscopic healing.^4^ However, the IOIBD acknowledges that these thresholds are largely unvalidated and may not predict clinical outcomes. While > 50% decrease in SES-CD has been shown to be associated with steroid-free remission,^17^ its linear scoring system that equally weights disease presence and severity in all areas of the terminal ileum and colon fails to capture the relative impact of each parameter on achieving EH. Indeed, Narula et al. found that significant ulceration in the ileum and rectum was associated with reduced odds of achieving endoscopic healing compared to similar disease burden in other areas of the colon, a difference that is not captured by the SES-CD.^8^ The MM-SES-CD’s strength as a scoring tool lies in its ability to capture this heterogeneity in disease burden and more accurately prognosticate EH. In this study, we found that patients with moderate (MM-SES-CD 31-44) or severe (MM-SES-CD ≥ 45) disease had significantly lower odds of achieving endoscopic healing (MM-SES-CD < 22.5) compared to those with mild disease. Although a previous cutoff for mild endoscopic disease was proposed as MM-SES-CD scores ≥ 14 to < 31, this was redefined in light of subsequent evidence suggesting a proposed target for endoscopic healing as MM-SES-CD < 22.5. Our results reinforce the finding that baseline endoscopic disease severity as measured by the MM-SES-CD predicts 1-year EH, as a previous study found that 30.3% of patients with mild endoscopic disease at baseline (MM-SES-CD 14-31) achieved endoscopic remission (SES-CD < 3) at 1-year compared to 8.8% of patients with severe disease (MM-SES-CD ≥ 45).^11^

In this post-hoc analysis, 163 (42.2%) patients out of 386 were excluded due to baseline MM-SES-CD scores < 22.5. Of note, the SEAVUE trial included 134 (34.7%) patients classified as having mild (SES-CD 3-6) endoscopic disease.^14^ Previous analyses have shown that MM-SES-CD < 22.5 has a similar predictive value for disease progression compared to SES-CD < 3 (AUC 0.81, 95% CI 0.68-0.94 vs AUC 0.75, 95% CI 0.62-0.87), the latter of which has been used by the International Organization of Inflammatory Bowel Diseases (IOIBD) to define endoscopic healing.^13^ This is worth highlighting given that many patients included within the SEAVUE trial had mild to inactive disease according to the MM-SES-CD, and likely this has some ramifications when it comes to clinical trial outcomes. Indeed, Narula et al conducted a post-hoc analysis using SEAVUE data and found among participants with a baseline MM-SES-CD ≥ 31 (moderate to severe disease), more patients treated with ustekinumab achieved endoscopic healing (MM-SES-CD < 22.5) and clinical remission compared to adalimumab, a conclusion that differs from that of the original trial.^18^ It is plausible that the use of the MM-SES-CD to measure endoscopic activity as baseline inclusion criteria could create a different study population with potentially different results observed as compared to currently accepted SES-CD-based criteria.

The MM-SES-CD may also have utility in clinical practice. Compared to the SES-CD, the MM-SES-CD better accounts for the differential impact of disease location on attaining EH, allowing clinicians to adjust their expectations and treatment plans accordingly. Narula et al. have previously shown that a baseline MM-SES-CD score >45 (indicative of severe disease) corresponds to a low probability of achieving ER at 1 year (6.7% in the testing cohort).^11^ Our results support these findings, suggesting that higher baseline MM-SES-CD scores correlate with lower odds of achieving endoscopic healing and FCP remission at 1 year. Although the SEAVUE cohort only included biologic-naïve patients with Crohn’s disease, two of the three studies that made up the testing cohort for the MM-SES-CD included patients who had previously failed tumor necrosis factor (TNF) antagonists,^11^ suggesting our findings may be applicable to bio-naïve and bio-experienced patients with CD. Clinicians may utilize this information when determining who needs more aggressive management of CD (eg, consideration of combination therapy or surgery over monotherapy), and set patients’ expectations regarding their likelihood of achieving clinical and endoscopic healing accordingly. Conversely, patients with low MM-SES-CD scores (<22.5) may be reassured that their disease is adequately controlled, even if they may have a few erosions that persist on colonoscopy. Interestingly, this threshold for EH includes patients who would be considered to have mild-moderate disease per the SES-CD (eg, patients with ileal disease only). While not assessed in this study, we have also previously shown that achieving MM-SES-CD <22.5 while on active therapy is associated with a low probability of long-term disease progression.^13^ Future validation studies of the MM-SES-CD using bio-experienced cohorts are also warranted. Finally, although baseline MM-SES-CD is predictive of 1-year clinical, biochemical, and endoscopic remission, model characteristics do suggest that even patients with high MM-SES-CD scores can achieve these outcomes, but albeit just with significantly lower probability than those with lower MM-SES-CD scores. Thus, other factors (eg, disease duration, number of advanced therapy failures, etc.) in addition to MM-SES-CD should be taken into consideration when trying to prognosticate a patient’s likelihood of achieving clinical, endoscopic, and biochemical success when initiating biologic therapies.

Overall, the MM-SES-CD represents a powerful tool that may be used to prognosticate outcomes of patients on biologic therapy, allowing providers to tailor their treatment decisions and patient counseling based on the likelihood of a favorable outcome. Its broader use may also allow for greater tolerance of small amounts of residual disease, preventing unnecessary treatment escalation.

This study’s strengths include utilizing a high-quality clinical trial dataset that utilized endoscopic evaluations assessed by blinded central readers. In addition to validating MM-SES-CD cutoffs for disease severity and their association with 1-year EH and PRO2 remission, we also showed an association between baseline MM-SES-CD score and FCP remission, which has not previously been described. Finally, though not the focus of this study, the MM-SES-CD shows promise as a tool for selecting relevant patient populations for future clinical trials. However, this work is not without limitations. Most importantly, this validation cohort (SEAVUE) and the derivation cohorts utilized to create the MM-SES-CD (the EXTEND, UNITI, and CT-P13 studies)^10^ were trials investigating the effect of biologic therapies in both biologic-naïve (SEAVUE, CT-P13 study) and mixed cohorts of biologic-naïve and experienced (EXTEND, UNITI) patients. It is unclear whether the thresholds described here accurately predict EH, PRO2 remission, and FCP remission in patients using non-biologic based therapies or in those who have failed multiple advanced therapies Future studies addressing the utility of the MM-SES-CD in certain subpopulations of patients with CD (eg, those not on biologics or those who have failed multiple biologics) are warranted. Furthermore, previous analyses have suggested MM-SES-CD ileal disease could also be categorized and was predictive of 1-year EH,^11^ but this was not assessed for in this validation analysis due to low sample size. We chose to exclude patients with low baseline MM-SES-CD scores as these patients were deemed unlikely to have any meaningful change in EH regardless of the treatment used. However, this may have introduced an element of selection bias to our findings, such that our findings may be less applicable to patients with quiescent disease. Finally, the AUC for the MM-SES-CD falls below the ideal cutoff of 0.8, somewhat limiting its predictive value. While baseline MM-SES-CD scores are predictive of endoscopic, clinical, and biochemical remission at 1 year, this limitation of the model should be considered and other patient factors taken into account.

## Conclusion

Our study demonstrates that baseline MM-SES-CD-based cutoffs for endoscopic disease severity have prognostic value for determining the likelihood of achieving endoscopic healing, PRO2 remission, and fecal calprotectin remission at 1 year in patients with CD. The MM-SES-CD should be considered for the evaluation of endoscopic activity in Crohn’s disease for both future trials and clinical practice.

## Supplementary Material

izaf137_Supplementary_Material

## Data Availability

Data will be shared upon request to the corresponding author with permission of YODA.
