# Supplementary material for: Validation of the Modified Multiplier of SES-CD (MM-SES-CD) to Predict Endoscopic Healing in Crohn’s Disease: A Post Hoc Analysis of the SEAVUE Trial
Source: Inflamm Bowel Dis. 2025 Jun 30;31(11):3103–11. doi: 10.1093/ibd/izaf137 (PMC12638049; doi:10.1093/ibd/izaf137)

**Supplementary Table 1:** MM-SES-CD modifiers and parameters

| **Segment** | **Baseline SES-CD Parameter** | **Category** | **SES-CD Score** | **Multiplier** | **Total** |
| --- | --- | --- | --- | --- | --- |
| Segment | Number of segments with ulcerations | 0  1  2  3  4  5 | N/A | x 4 | 0  4  8  12  16  20 |
| Ileum | Presence of ulcerations | None  Small  Medium  Large | 0  1  2  3 | x 1 | 0  1  2  3 |
|  | Extent of ulcerations | 0%  <10%  10-30%  >30% | 0  1  2  3 | x 3 | 0  3  6  9 |
|  | Affected surface | 0%  <50%  50-75%  >75% | 0  1  2  3 | x 5 | 0  5  10  15 |
|  | Presence of strictures | None  Single, passable  Multiple, passable  Non-passable | 0  1  2  3 | x 4 | 0  4  8  12 |
| Right Colon | Presence of ulcerations | None  Small  Medium  Large | 0  1  2  3 | x 3 | 0  3  6  9 |
|  | Extent of ulcerations | 0%  <10%  10-30%  >30% | 0  1  2  3 | x 2 | 0  2  4  6 |
|  | Affected surface | 0%  <50%  50-75%  >75% | 0  1  2  3 | x 1 | 0  1  2  3 |
|  | Presence of strictures | None  Single, passable  Multiple, passable  Non-passable | 0  1  2  3 | x 1 | 0  1  2  3 |
| Transverse colon | Presence of ulcerations | None  Small  Medium  Large | 0  1  2  3 | x 1 | 0  1  2  3 |
|  | Extent of ulcerations | 0%  <10%  10-30%  >30% | 0  1  2  3 | x 1 | 0  1  2  3 |
|  | Affected surface | 0%  <50%  50-75%  >75% | 0  1  2  3 | x 1 | 0  1  2  3 |
|  | Presence of strictures | None  Single, passable  Multiple, passable  Non-passable | 0  1  2  3 | x 1 | 0  1  2  3 |
| Left Colon | Presence of ulcerations | None  Small  Medium  Large | 0  1  2  3 | x 3 | 0  3  6  9 |
|  | Extent of ulcerations | 0%  <10%  10-30%  >30% | 0  1  2  3 | x 2 | 0  2  4  6 |
|  | Affected surface | 0%  <50%  50-75%  >75% | 0  1  2  3 | x 1 | 0  1  2  3 |
|  | Presence of strictures | None  Single, passable  Multiple, passable  Non-passable | 0  1  2  3 | x 2 | 0  2  4  6 |
| Rectum | Presence of ulcerations | None  Small  Medium  Large | 0  1  2  3 | x 3 | 0  3  6  9 |
|  | Extent of ulcerations | 0%  <10%  10-30%  >30% | 0  1  2  3 | x 1 | 0  1  2  3 |
|  | Affected surface | 0%  <50%  50-75%  >75% | 0  1  2  3 | x 0.5 | 0  0.5  1  1.5 |
|  | Presence of strictures | None  Single, passable  Multiple, passable  Non-passable | 0  1  2  3 | x 2 | 0  2  4  6 |

**Supplementary Table 2 -** Univariate analysis of baseline variables and association with one-year endoscopic healing (MM-SES-CD < 22.5)

|  | OR | 95% CI | P- value |
| --- | --- | --- | --- |
| **Univariate analysis** | | | |
| Mild (MMSESCD ≥22.5 and < 31) | Reference | Reference | Reference |
| Moderate (MMSESCD ≥ 31 and <45) | 0.39 | 0.16 – 0.92 | 0.03 |
| Severe (MMSESCD ≥45) | 0.17 | 0.07 – 0.38 | < 0.001 |
| Age | 1.01 | 0.99 – 1.04 | 0.46 |
| Sex (males) | 0.61 | 0.32 – 1.14 | 0.12 |
| Race: Asian White Black Others | Reference 0.97 0.90 1.20 | Reference  0.19 – 4.10 0.09 – 10.09 0.08 – 32.7 | Reference  0.966 0.928  0.89 |
| Duration in years | 1.00 | 0.96 – 1.05 | 0.99 |
| Prior Surgery | 1.09 | 0.53 – 2.31 | 0.81 |
| Location: Colonic Ileal Ileocolonic | Reference 2.39 1.97 | Reference 0.88 – 6.76 0.86 – 4.55 | Reference 0.09 0.11 |
| Perianal disease | 0.74 | 0.37 – 1.48 | 0.39 |
| Active intervention: Adalimumab Ustekinumab | Reference 1.61 | Reference 0.86 – 3.04 | Reference 0.14 |
| Upper GI disease | 0.38 | 0.15 – 0.91 | 0.03 |
| Baseline steroid usage | 0.65 | 0.25 – 1.74 | 0.38 |
| Baseline IMM usage | 1.6 | 0.65 – 4.36 | 0.33 |
| Current smoking |  |  |  |
| Baseline Hb | 1.03 | 0.86 – 1.25 | 0.72 |
| Baseline albumin (normal > 3.5 g/dL) | 1.98 | 0.93 – 4.36 | 0.08 |
| Baseline CRP (normal: < 5 mg/dL) | 1.0008 | 0.99 – 1.02 | 0.89 |
| Baseline FCP (normal: <250 mcg/kg) | 0.99 | 0.99 – 0.99 | 0.03 |

**Supplementary Table 3:** Association between baseline MM-SES-CD and one-year SES-CD based remission (SES-CD <3), n=165

|  | Odds Ratios | P- value |
| --- | --- | --- |
| Modified MM-SESCD (mild) | 1 (reference) |  |
| Modified MM-SESCD (moderate) | 0.74 (0.31 – 1.78) | 0.51 |
| Modified MM-SESCD (severe) | 0.49 (0.19 – 1.22) | 0.13 |

**Supplementary Table 4**: Association between baseline MM-SES-CD score and one-year fecal calprotectin remission, n=141

|  | OR | 95% CI | P- value |
| --- | --- | --- | --- |
| **Univariate analysis** | | | |
| Mild (MMSESCD ≥22.5 and < 31) | Reference | Reference | Reference |
| Moderate (MMSESCD ≥ 31 and <45) | 0.54 | 0.22 – 1.29 | 0.17 |
| Severe (MMSESCD ≥45) | 0.36 | 0.15 – 0.85 | 0.02 |

**Supplementary Figure 1**: Percentage of patients treated with adalimumab who achieved MM-SES-CD-based endoscopic healing (score <22.5) at one-year, stratified by baseline MM-SES-CD score categories (mild ≥ 22.5 to < 31, moderate ≥ 31 to < 45, severe ≥ 45)


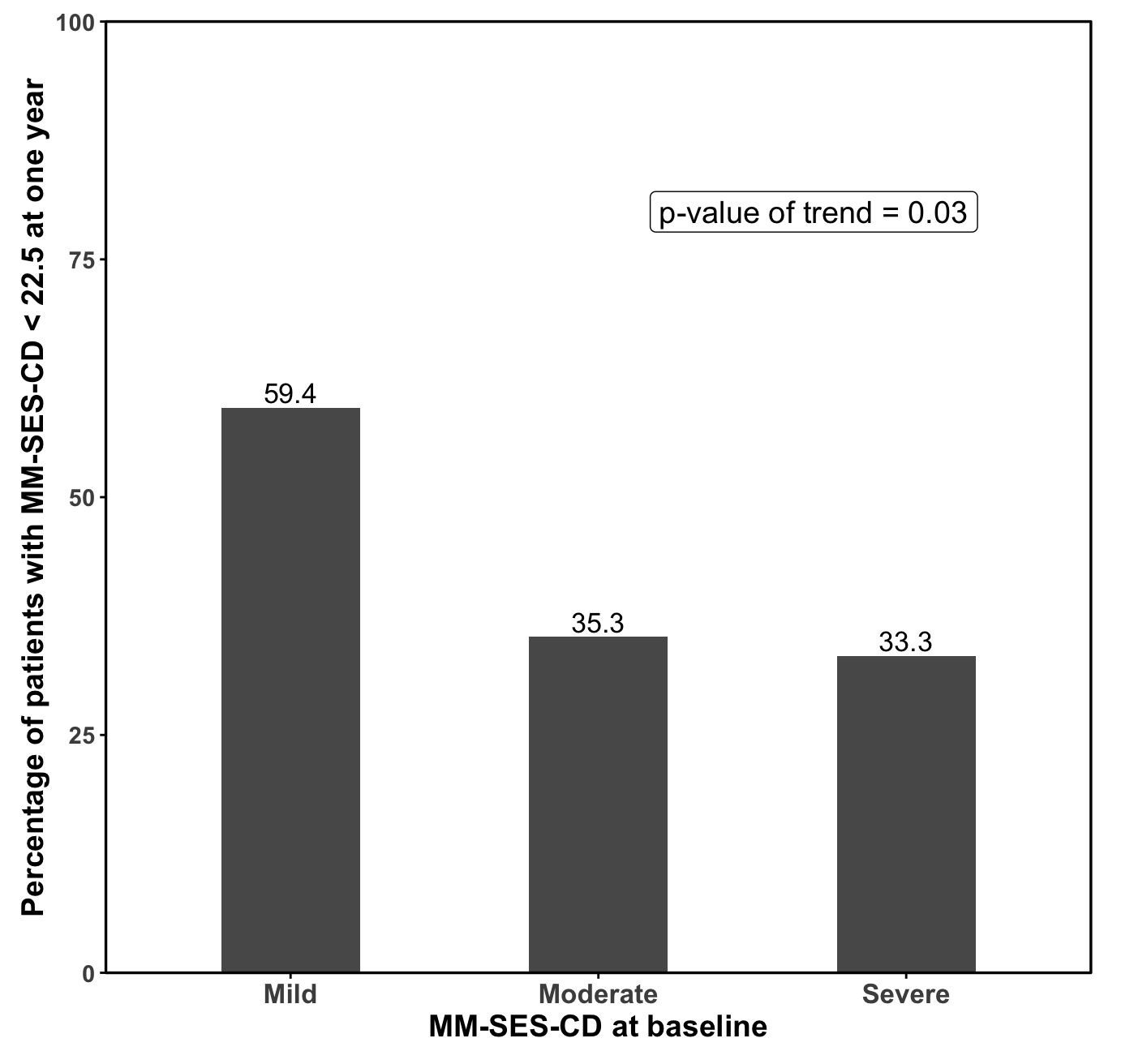


**Supplementary Figure 2:** Percentage of patients treated with ustekinumab who achieved MM-SES-CD-based endoscopic healing (score <22.5) at one-year, stratified by baseline MM-SES-CD score categories (mild ≥ 22.5 to < 31, moderate ≥ 31 to < 45, severe ≥ 45)


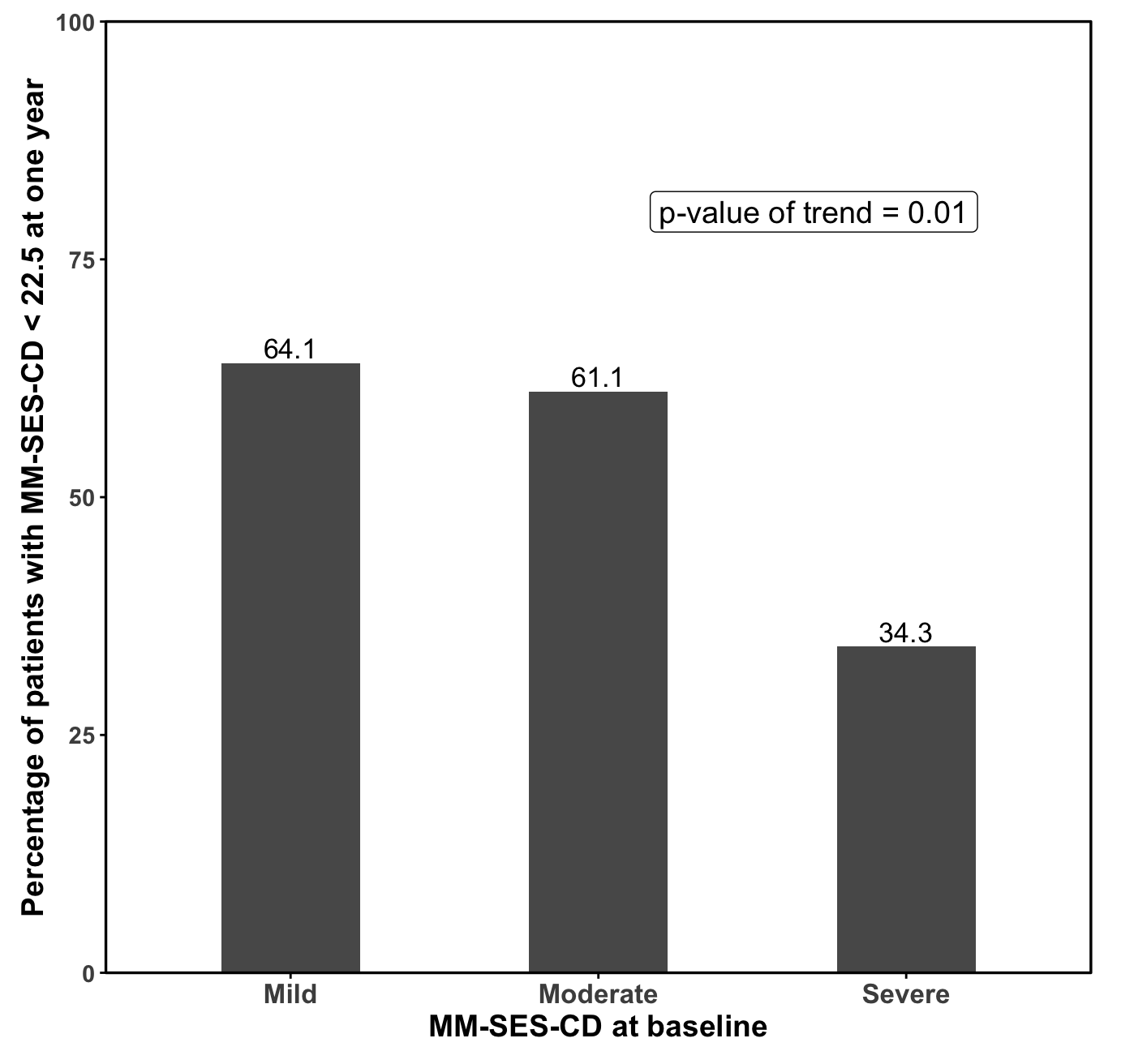


**Supplementary Figure 3:** Percentage of patients in SES-CD remission (<3) at one-year stratified by baseline MM-SES-CD categories (mild ≥ 22.5 to < 31, moderate ≥ 31 to < 45, severe ≥ 45


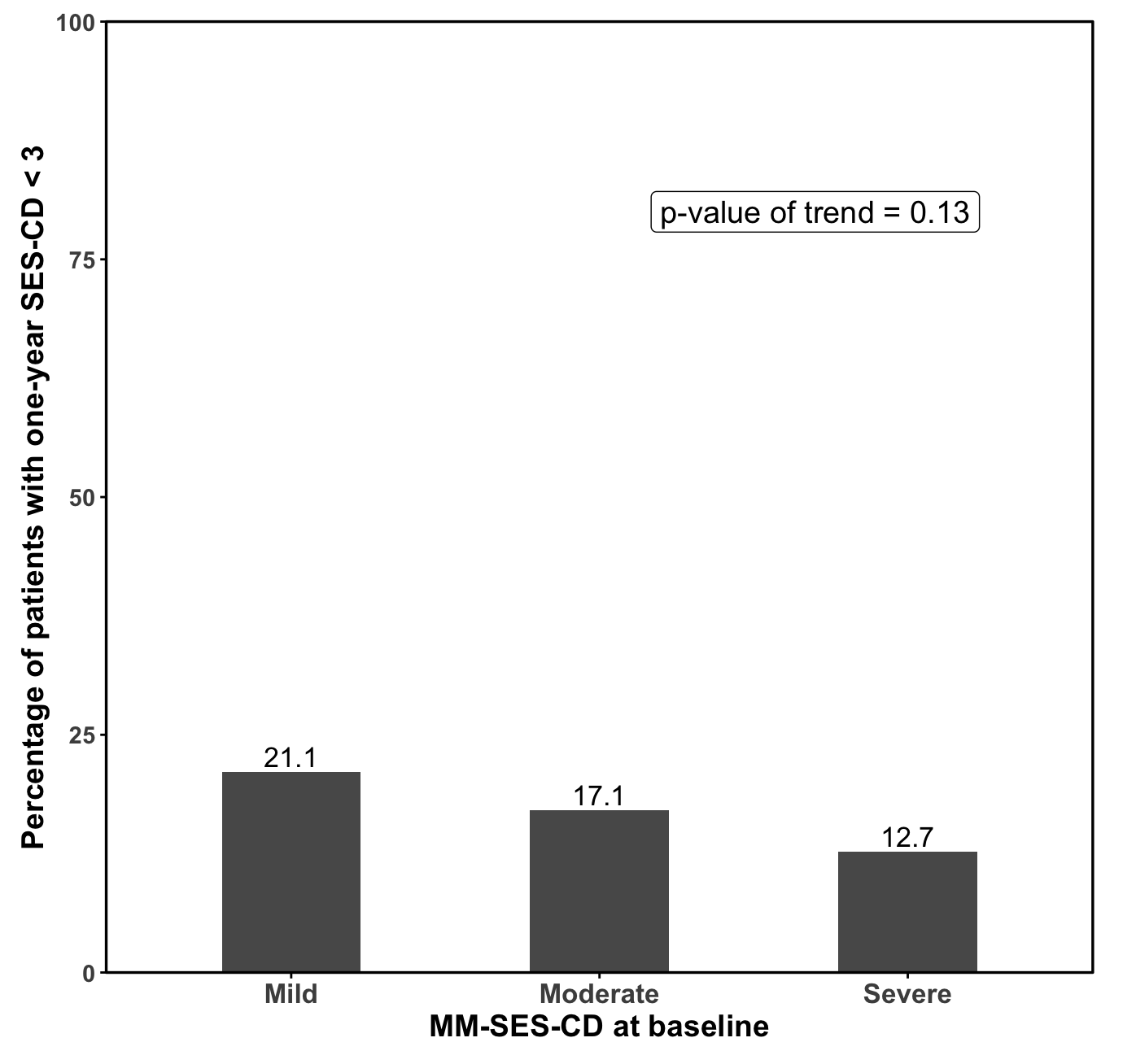

Supplement: izaf137_Supplementary_Material [file izaf137_supplementary_material.docx]
